# Simulation-based evaluation of the impact of dose fractionation study design on antibiotic PKPD analyses

**DOI:** 10.1093/jacamr/dlaf057

**Published:** 2025-04-11

**Authors:** Raphaël Saporta, Muskan Madan, Lena E Friberg

**Affiliations:** Department of Pharmacy, Uppsala University, Uppsala, Sweden; Department of Pharmacy, Uppsala University, Uppsala, Sweden; Department of Pharmacy, Uppsala University, Uppsala, Sweden

## Abstract

**Objectives:**

To evaluate the impact of antibiotic dose fractionation study design on pharmacokinetic/pharmacodynamic (PK/PD) indices and PKPD model estimation.

**Methods:**

PKPD models for meropenem and polymyxin B (PMB) were applied to (i) simulate various dose fractionation studies in mice to derive PK/PD indices and efficacy targets and (ii) perform stochastic simulations and estimations evaluating which efficacy assessment times, in addition to 24 h, would improve the estimation of drug effect parameters.

**Results:**

The *R*^2^ values of PK/PD indices were primarily influenced by reductions of the dosing intervals for meropenem and by decreases of the lowest total daily dose for PMB. For certain study designs (e.g. frequent administration of higher meropenem doses), *R*^2^ values for *f*T > MIC and *f*AUC/MIC were similar. Efficacy target magnitudes were also sensitive to the selected doses. Additional efficacy assessment times improved parameter accuracy (e.g. 40% reduction in relative root mean squared error of PMB effect slope). The model parameter accuracy was more affected by the selection of time points for meropenem, which included resistance, than for PMB. Efficacy measurements in the first hours after treatment start (e.g. 2 and 6 h), in addition to 24 h, were essential for resistance characterization.

**Conclusions:**

The choice of doses and fractionations impacted PK/PD index selection and efficacy target magnitude. Depending on the antibiotic, the dose or fractionation selection appeared to be the most critical. Early treatment efficacy measurements were beneficial to PKPD model-based analyses, particularly to describe resistance processes.

## Introduction

Understanding an antibiotic’s pharmacokinetics (PK) and pharmacodynamics (PD) is pivotal in drug development for selecting a clinical dose effective against bacterial infections. Preclinical PKPD studies, required by authorities, are often key to support the choice of dose.^1,2^  *In vitro* and *in vivo* experimental systems are commonly used for PKPD studies in antibiotic drug development.^3,4^ The minimum inhibitory concentration (MIC) is assayed *in vitro* to evaluate the antibiotic activity against a defined bacterial strain. The MIC, however, possesses limitations, as it only represents an evaluation of growth prevention at one time point, thus ignoring the time course of bacterial dynamics. The MIC is also subject to biological and assay variation.^5–7^ The antibiotic PKPD is typically studied *in vivo* using neutropenic mouse thigh or lung infection models.^3,4,8^ Nonetheless, the results of PKPD analyses may be affected by the chosen study design to an extent that is not fully known.

A common approach is using PK/PD indices to guide antibiotic dose selection.^9^ PK/PD indices link a summary metric of the antibiotic PK to the MIC as a marker of antibiotic PD. Three main PK/PD indices are typically evaluated: the maximum concentration (C_max_) to MIC ratio (C_max_/MIC), the AUC to MIC ratio (AUC/MIC) and the fraction of time concentrations exceed the MIC (T > MIC), all usually evaluated using unbound (free) antibiotic concentrations (*f*C_max_/MIC, *f*AUC/MIC, *f*T > MIC). PK/PD indices are investigated for their correlation with efficacy, which is evaluated by determining the bacterial count in the infected tissue, typically 24 h after the start of treatment. The overall aim of such analyses is to identify the PK/PD index that is most correlated with antibiotic efficacy and to derive PK/PD targets, which are the magnitude of a PK/PD index required to reach a specific efficacy (such as bacterial stasis, 1-log or 2-log kill). PK/PD indices have limitations, as they share limitations of the MIC, rely on summary metrics of PK, and evaluate efficacy at only one time point.^6,10^ Model-based approaches have been suggested as a more informative alternative to PK/PD indices.^10,11^ PKPD models can describe the impact of an antibiotic’s concentration-time profile on the time course of bacterial growth and killing while accounting for the presence of resistant bacteria.^12,13^

Dose fractionation studies are often performed for *in vivo* PKPD analyses, in which a total antibiotic dose is divided into multiple doses administered at different dosing intervals. Such designs allow for variations in C_max_ and T > MIC for the same total dose, contributing to understanding antibiotic PKPD and PK/PD index selection. Nonetheless, PK/PD index selection and target PK/PD magnitude can be sensitive to experimental design (e.g. frequent administrations leading to selecting *f*AUC/MIC over *f*T > MIC).^14^ Still, the extent of the design’s impact on such analyses remains unclear. Furthermore, as a dose fractionation study design typically evaluates efficacy solely at 24 h after the start of treatment, these studies provide limited information for developing PKPD models describing time courses of bacterial growth and killing. The current study aimed to evaluate further the impact of the antibiotics’ dose fractionation study design on the determination of PK/PD indices for PK/PD index-based analyses, and on the estimation of parameters for PKPD model-based analyses. Simulations of antibiotic dose fractionation studies with various designs were performed using literature PKPD models to derive PK/PD indices and efficacy targets. A simulation and estimation methodology was applied to evaluate which additional time points for evaluation of antibiotic efficacy in a dose fractionation study would be most informative for the estimation of PKPD model parameters.

## Materials and methods

### PKPD models

Previously developed PKPD models were used for two antibiotics of different classes: meropenem, a β-lactam typically associated with a ‘time-dependent’ effect best described by *f*T > MIC,^15^ and polymyxin B (PMB), which is usually associated with a ‘concentration-dependent’ effect best described by *f*AUC/MIC.^16,17^

For meropenem, a one-compartment model with first-order absorption^18^ described the plasma concentrations. Simulated unbound concentrations drove the bacterial killing in a PKPD model for meropenem developed based on *in vitro* time-kill data.^19^ The parameters estimated for the *Pseudomonas aeruginosa* strain ATCC 27853 (MIC = 1 mg/L) were applied. These models have earlier been demonstrated to predict the results of an *in vivo* dose fractionation study in a simulation-based analysis.^14^ The PKPD model describes two bacterial subpopulations: a susceptible (subpopulation 1) and a pre-existing resistant (subpopulation 2) subpopulation, with a fraction of bacteria (Mut) in subpopulation 2 in the start inocula. Bacteria from both subpopulations may be in either a drug-susceptible and growing (S) state or a non-susceptible, non-growing, dormant (D) state. The meropenem killing rate constant against ATCC 27853 was described by a power effect model, in which higher concentrations were required to reach the same effect in the resistant subpopulation as in the susceptible subpopulation:


(1) $$\begin{array}{c} k_{\mathrm{drug},1}=C^{\gamma }\cdot \mathrm{Slope}\; \end{array}$$

(2) $$\begin{array}{c} k_{\mathrm{drug},2}={\left(\frac{C}{\mathrm{Shift}}\right)}^{\gamma }\cdot \mathrm{Slope}\; \end{array}$$

where *C* is the unbound (fu = 0.81)^18^ drug concentration, *k*_drug,1_ and *k*_drug,2_ the drug effect rate constants for subpopulations 1 and 2, respectively, γ is the Hill factor, Shift the estimated concentration shift for the resistant subpopulation relative to the susceptible population, and Slope the drug effect slope.

For PMB, PK and PKPD models based on neutropenic mouse thigh infection data were used.^20^ A two-compartment model with saturable absorption and linear elimination described PMB PK. Unbound concentrations (fu = 0.166) were used to drive PMB efficacy against a clinical strain of *Acinetobacter baumannii* (CS01,^21^ MIC = 0.25 mg/L).^22^ Drug effect on the bacterial population was implemented as a power function (Eq. 1). The model also included a parameter accounting for an inoculum effect, which was not considered in this analysis as simulations were only performed for one inoculum size.

### Impact of dose fractionation design on PK/PD index analyses

Dose fractionation studies were simulated using the PKPD models, with an initial bacterial count of 6.5 log_10_ cfu/mL for meropenem (within the range of an earlier study),^23^ and 6 log_10_ cfu/thigh for PMB (median inoculum in the PKPD model).^20^ Simulations were performed without residual variability to mimic median experimental values in each group, assuming the number of animals per group would be sufficient to limit the impact of experimental variability on the PK/PD index analysis.

Dose fractionation studies with different designs, summarized in Table 1, were simulated to assess how the study design affects the PK/PD index analysis. A rich design with eight evaluated doses and eight fractionations was simulated to derive model-based reference PK/PD index values and targets. Then, earlier designs applied in dose fractionation studies were used as a basis to create study designs with variations in one or multiple design parameters.^14,16^ The number of evaluated doses and fractionations was the same in these scenarios (three total daily doses and four fractionations per dose). The alterations to the designs, selected to change the covered antibiotic exposure-response, were based on one or a combination of three main adjustments: a change to one of the total daily doses, a shift in the evaluated dose range, or a change in the selected dose fractionations. Drug concentrations and bacterial dynamics were simulated over 24 h of treatment.

**Table 1.** Summary of the dose fractionation study designs simulated for PK/PD index computation

Antibiotic	Design	Doses (mg/kg/day)	Fractionations (dosing interval in hours)	No of treatment groups
Meropenem	Rich	25, 100, 200, 400, 600, 800, 1000, 1200	1, 2, 3, 4, 6, 8, 12, 24	64
	Literature	200, 400, 800	3, 6, 12, 24	12
	Alteration	Lowest dose: 12.5 to 400	Four fractionations selected from: 1, 2, 3, 4, 6, 8, 12, 24	12
		Intermediate dose: 200 to 800		
		Highest dose: 400 to 2500		
Polymyxin B	Rich	0.5, 10, 22.5, 30, 45, 60, 90, 120^a^	1, 2, 3, 4, 6, 8, 12, 24	64
	Literature	22.5, 45, 90	4, 8, 12, 24	12
	Alteration	Lowest dose: 0.5 to 45	Four fractionations selected from: 1, 2, 3, 4, 6, 8, 12, 24	12
		Intermediate dose: 22.5 to 90		
		Highest dose: 45 to 120^a^		

Control groups at the start of treatment and 24 h after the start of treatment were included in every evaluated design. Alteration designs were based on changes to the literature design, in which one or multiple elements were modified. The altered designs’ range of evaluated doses and potential fractionations are reported.

^a^120 mg/kg/day was reported as the maximum tolerated polymyxin B dose in mice, higher daily doses were consequently not explored.^16^

Based on simulated dose fractionation studies, PK/PD indices were derived, and a sigmoid *E*_max_ was fitted to evaluate the relationship between PK/PD indices and response, defined as the bacterial counts at 24 h:


(3) $$\begin{array}{c} E=E_{0}-\;\frac{PD_{\max}\;\cdot \;\mathrm{Inde}x^{H}}{{{\mathrm{Index}}_{50}}^{H}+\mathrm{Inde}x^{H}} \end{array}$$

where *E* is the bacterial load at 24 h, *E*_0_ is the bacterial load at 24 h when Index = 0, PD_max_ is the maximum bacterial load reduction from *E*_0_ at 24 h, *H* is the Hill factor for sigmoidal shape, Index is the PK/PD index value, and Index_50_ is the PK/PD index value required to reach half of PD_max_. The highest correlation based on *R*^2^ values was used to drive PK/PD index selection. Additionally, PK/PD targets for bacterial stasis, 1-log kill and 2-log kill were derived for the different PK/PD indices based on the fitted *E*_max_ models.

### Impact of dose fractionation design on PKPD model estimation

A stochastic simulation and estimation (SSE) approach was used to evaluate the impact of bacterial count measurement time points, in addition to 24 h after start of treatment, on PKPD model estimation. Simulations were performed using the literature model parameters (Table S1, available as Supplementary data at *JAC-AMR* Online), with the doses and fractionation of the literature-based designs (Table 1). These simulations, with five mice per time point and group, as typically used for mouse infection models,^24^ included residual error terms for the bacterial counts. Treatment was initiated 2 h after infection, with the same inoculum sizes as used in the simulations described above for PK/PD index analyses. The simplest design mimicked a standard dose fraction design with measures of the bacterial count at the start of treatment (2 h) and at 24 h after the start of treatment (26 h) in both control and treatment groups. Alternative designs included cfu determination at one or two intermediate time points, in 2-h steps in the range between 2 and 22 h after the start of treatment. A rich design, including all evaluated time points, was also assessed as a reference. For each design, 500 dose fractionation studies were simulated.

Model parameters were estimated based on the simulated data. Both PK and bacteria-related parameters (e.g. growth rate) were fixed, assuming the availability of sufficient PK and growth control data. The bias and precision of the estimated drug effect and residual variability parameters were evaluated using the relative estimation error (REE), the relative bias, and the relative root mean squared error (RRMSE), given by:


(4) $$\begin{array}{c} \mathrm{RE}E_{i}=\frac{P_{\mathrm{est},i}-P_{\mathrm{true}}}{P_{\mathrm{true}}} \end{array}$$

(5) $$\begin{array}{c} \mathrm{Relative}\;\mathrm{bias}=\frac{1}{N}\overset{N}{\underset{i=\;1}{\sum }}\frac{P_{\mathrm{est},i}-P_{\mathrm{true}}}{P_{\mathrm{true}}} \end{array}$$

(6) $$\begin{array}{c} \mathrm{RRMSE}=\sqrt{\frac{1}{N}\overset{N}{\underset{i=\;1}{\sum }}\;{\left(\frac{P_{\mathrm{est},i}-P_{\mathrm{true}}}{P_{\mathrm{true}}}\right)}^{2}} \end{array}$$

where *N* denotes the number of simulated studies, *P*_est,*i*_ denotes the parameter value estimated based on the *i*th simulated study and *P*_true_ denotes the parameter value used for simulation.

### Software

PK/PD indices and bacterial counts were simulated in R version 4.3.1 using mrgsolve version 1.3.0,^25,26^ with subsequent curve fitting in R using the drc package.^27^ SSEs were performed in NONMEM 7.5.0,^28^ aided by Perl-speaks-NONMEM version 5.3.1,^29^ using the first-order conditional estimation method, with a log_10_-transform-both-sides approach and a log_10_-additive residual error.

## Results

### Impact of dose fractionation design on PK/PD index analyses

**Figure 1.**
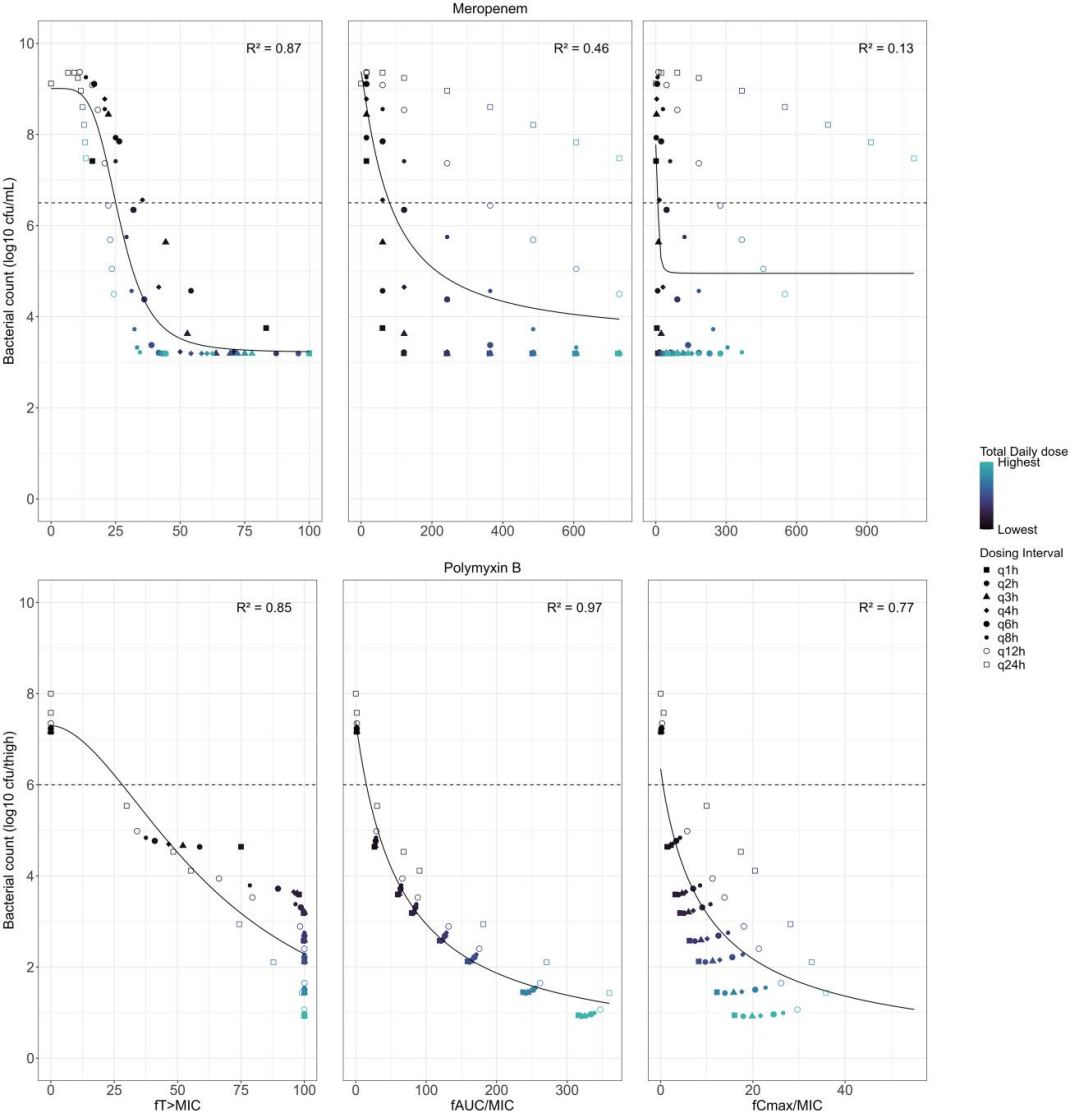
Relationship between the PK/PD indices and the antibiotic efficacy (bacterial count at 24 h) in mice for the rich dose fractionation designs of meropenem (top row) and polymyxin B (bottom row). The simulated bacterial counts at 24 h for the different dose groups (shapes), the stasis line (horizontal dashed line), and the fitted *E*_max_ curve (solid line) are shown. The *R*^2^ values of the curve fit are also indicated.

The simulated dose fractionation studies for the rich designs are presented in Figure 1. The PK/PD indices with the highest *R*^2^ values were *f*T > MIC for meropenem (*R*^2^ = 0.87) and *f*AUC/MIC for PMB (*R*^2^ = 0.97). PK/PD target values of *f*T > MIC for meropenem were determined at 25% for stasis, 29% for 1-log kill and 35% for 2-log kill. PK/PD target values of *f*AUC/MIC for PMB were determined at 15 for stasis, 31 for 1-log kill and 56 for 2-log kill.

**Figure 2.**
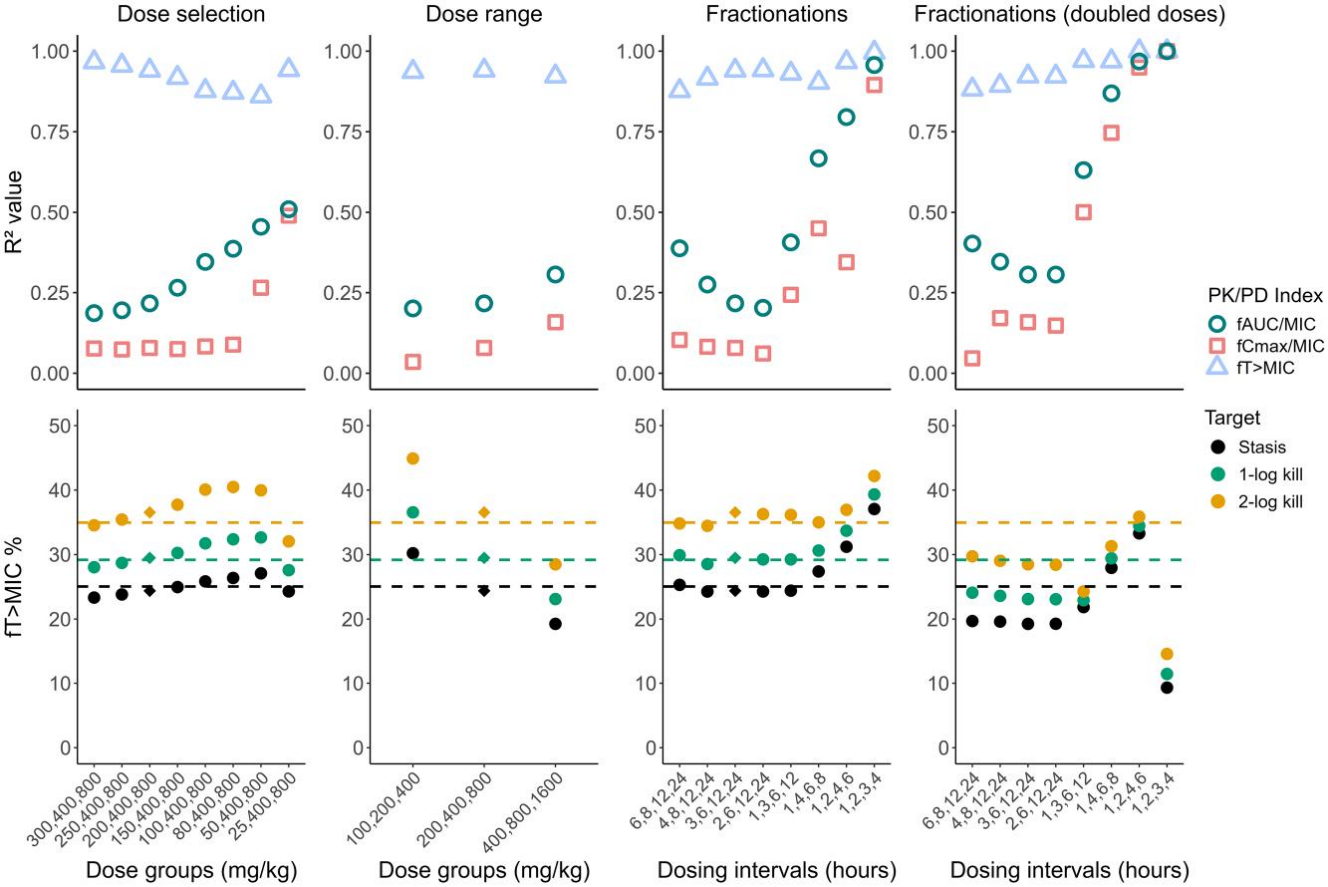
*R* ^2^ values for the different PK/PD indices (top row) and *f*T > MIC values required to achieve efficacy targets at 24 h (bottom row) based on *E*_max_ models fitted to simulated meropenem dose fractionation studies with different alterations to the study design (first column: changes to the lowest dose group, second column: changes to the dose range, third column: changes to the dose fractionations, fourth column: changes to the fractionations and doubled doses compared to the literature design). The literature design is marked with a diamond in the target panels. Horizontal dashed lines correspond to the *f*T > MIC target values identified with the rich design.

**Figure 3.**
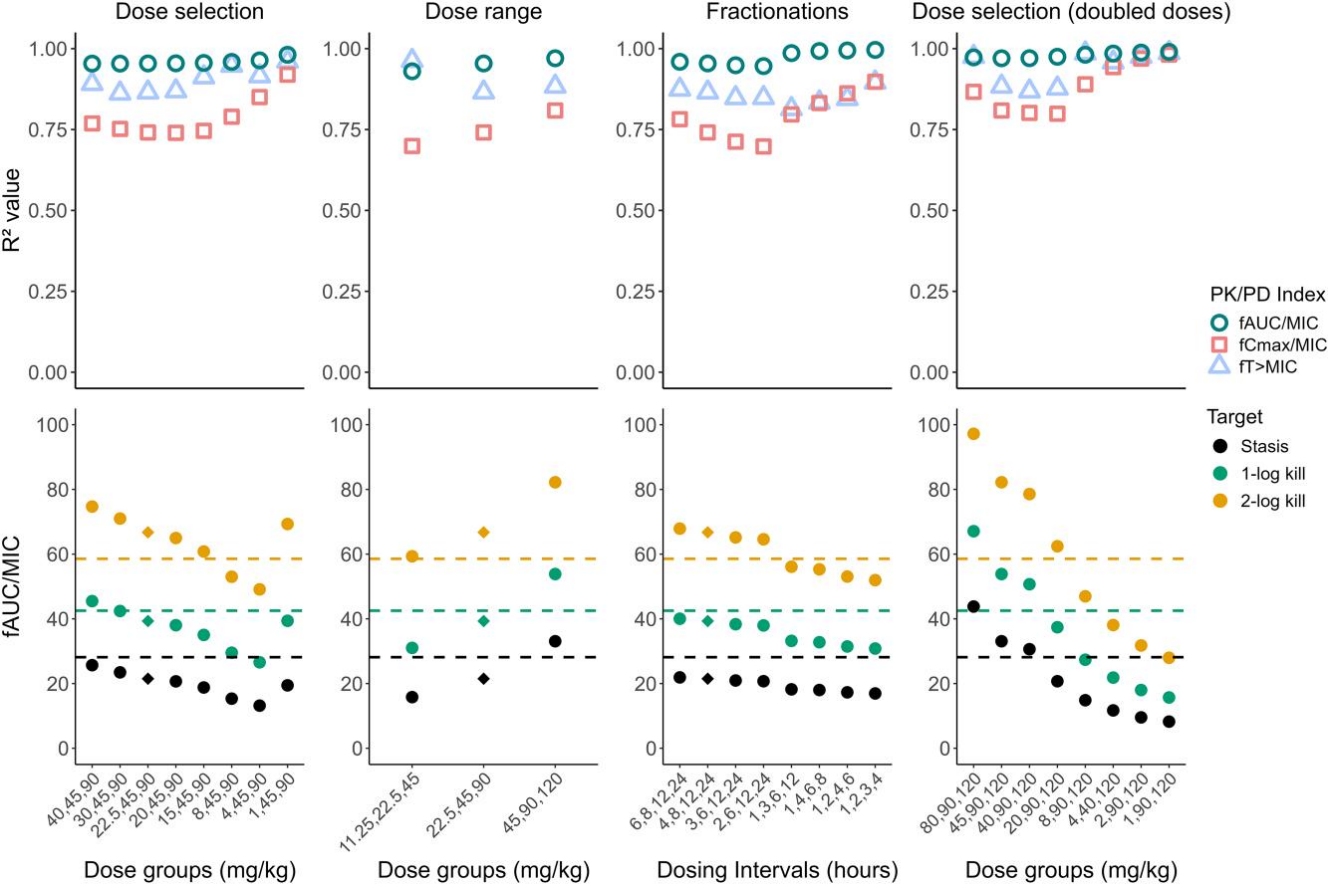
*R* ^2^ values for the different PK/PD indices (top row) and *f*AUC/MIC values required to achieve efficacy targets at 24 h (bottom row) based on *E*_max_ models fitted to simulated polymyxin B dose fractionation studies with different alterations to the study design (first column: changes to the lowest dose group, second column: changes to the dose range, third column: changes to the dose fractionations, fourth column: changes to the lowest dose group and doubled doses compared to the literature design). The literature design is marked with a diamond in the target panels. Horizontal dashed lines correspond to the *f*T > MIC target values identified with the rich design.

*R* ^2^ values and PK/PD target values are presented for some study designs of interest in Figures 2 and 3 for meropenem and PMB, respectively, and for all investigated designs in Tables S2 and S3. Meropenem appeared most affected by the selection of fractionations, with higher *R*^2^ for *f*AUC/MIC and *f*C_max_/MIC and higher *f*T > MIC target values when administrations were more frequent. PMB was most influenced by dose selection, with higher *R*^2^ values for *f*T > MIC and *f*C_max_/MIC when the lowest dose was reduced, while *f*AUC/MIC target values first decreased and then increased as the lower doses were reduced. For both antibiotics, the evaluated dose range impacted the PK/PD target magnitudes, with increased *f*AUC/MIC targets for PMB and decreased *f*T > MIC targets for meropenem with increased doses. For both antibiotics, *R*^2^ and PK/PD targets were further impacted by combinations of changes to the design. Overall, *f*T > MIC and *f*AUC/MIC remained the indices with the highest *R*^2^ values in most designs for meropenem and PMB, respectively.

### Impact of dose fractionation design on PKPD model estimation

**Figure 4.**
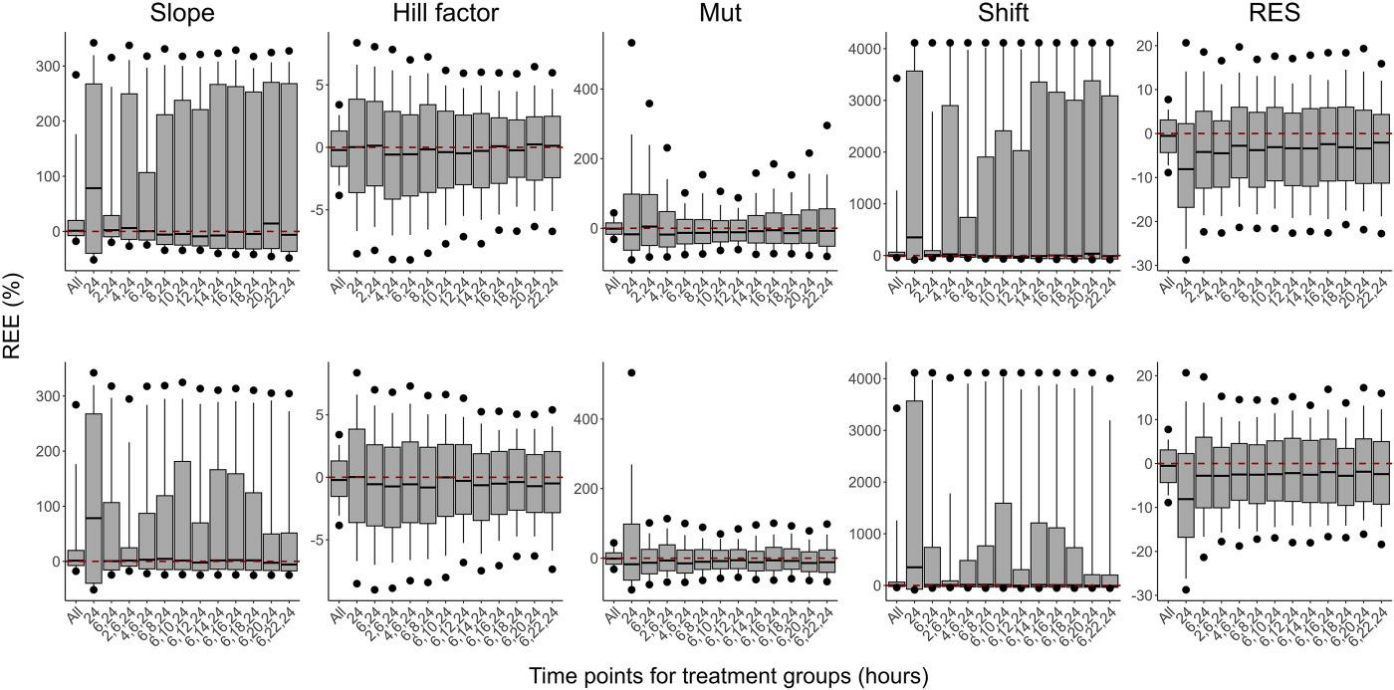
Relative estimation errors (REEs) of parameter estimates for meropenem for scenarios evaluating the inclusion of one (top row) or two (bottom row) extra time points (in hours after the start of treatment) for the evaluation of bacterial count in treatment groups. Shown are the median (solid line), 25th and 75th percentiles (box), 10th and 90th percentiles (whiskers) and the 5th and 95th percentiles (circles) of REEs from 500 simulations and estimations per scenario. Each column represents REEs for one parameter. Scenarios are indicated based on which time points were included in the design, with ‘All’ being the scenario with all evaluated time points and ‘24’ the simplest design with an effect evaluation at 24 h after the start of treatment only.

**Figure 5.**
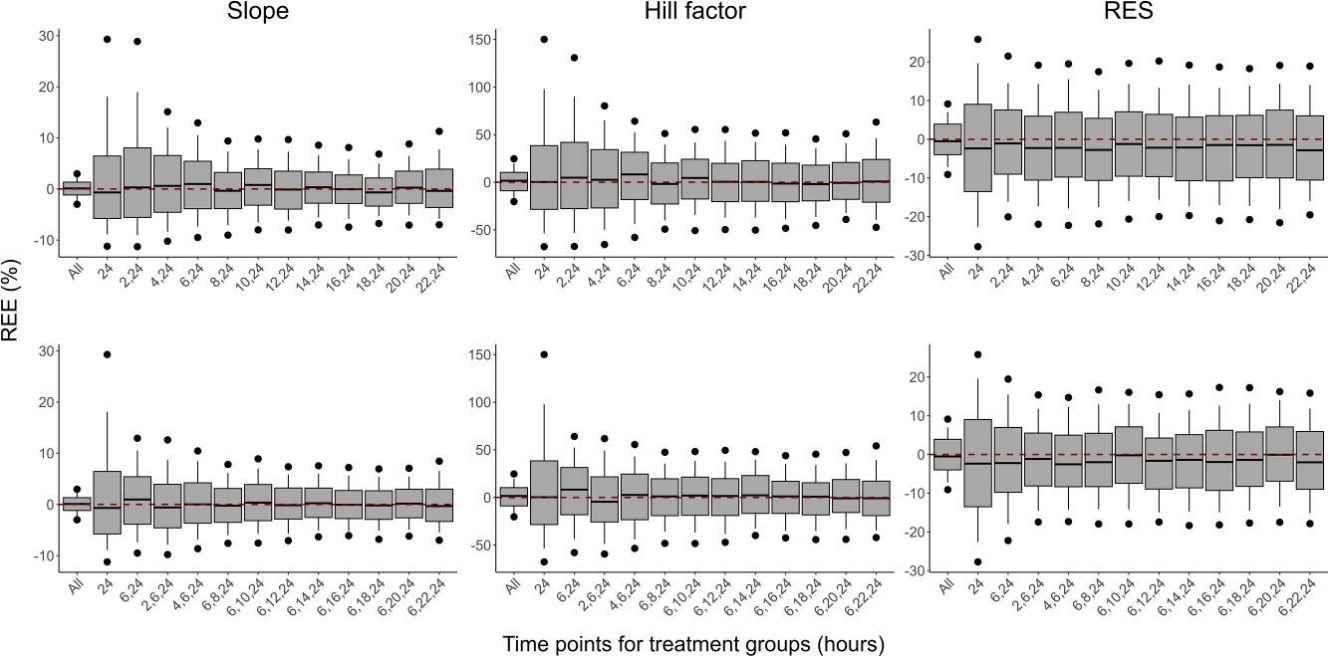
Relative estimation errors (REEs) of parameter estimates for polymyxin B for scenarios evaluating the inclusion of one (top row) or two (bottom row) extra time points (in hours after the start of treatment) for the evaluation of bacterial count in treatment groups. Shown are the median (solid line), 25th and 75th percentiles (box), 10th and 90th percentiles (whiskers) and the 5th and 95th percentiles (circles) of REEs from 500 simulations and estimations per scenario. Each column represents REEs for one parameter. Scenarios are indicated based on which time points were included in the design, with ‘All’ being the scenario with all evaluated time points and ‘24’ the simplest design with an effect evaluation at 24 h after the start of treatment only.

**Figure 6.**
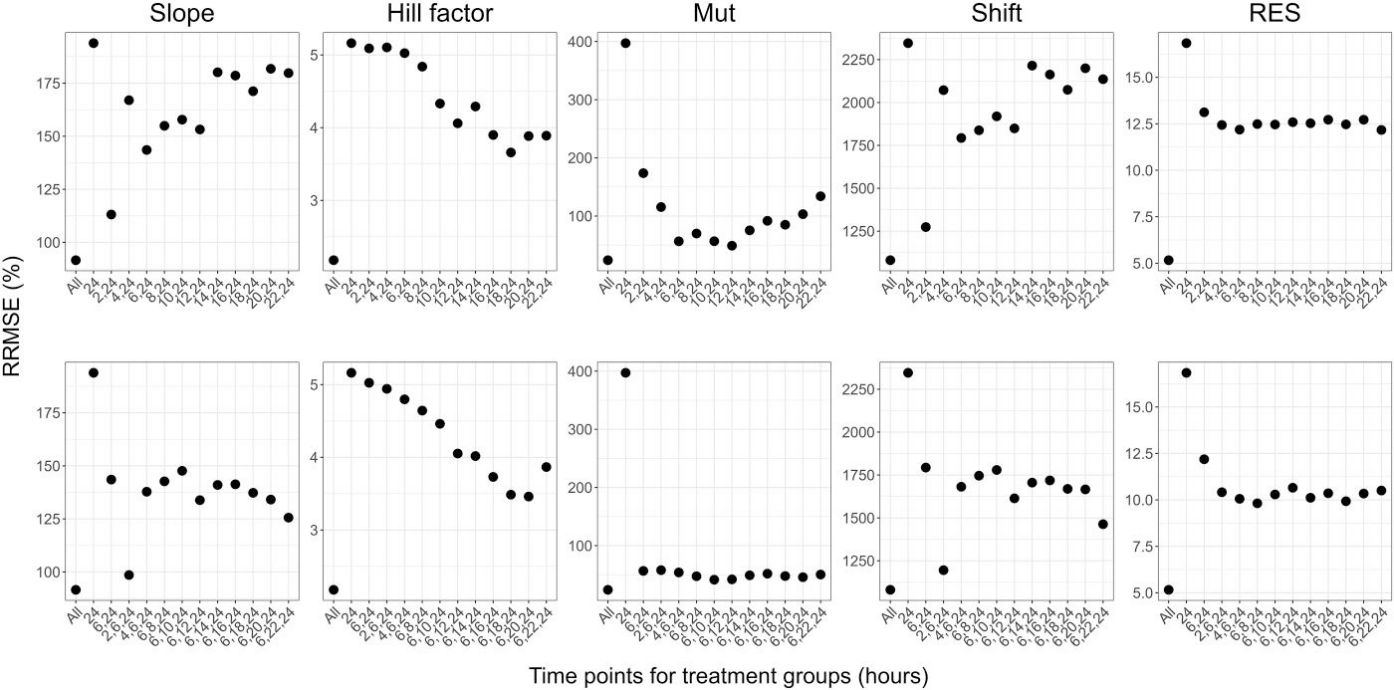
RRMSEs of parameter estimates for meropenem for scenarios evaluating the inclusion of one (top row) or two (bottom row) extra time points (in hours after the start of treatment) for the evaluation of bacterial count in treatment groups. Each column presents RRMSEs for one parameter. Scenarios are indicated based on which time points were included in the design, with ‘All’ the scenario with all evaluated time points, and ‘24’ the simplest design with an effect evaluation at 24 h after the start of treatment only.

**Figure 7.**
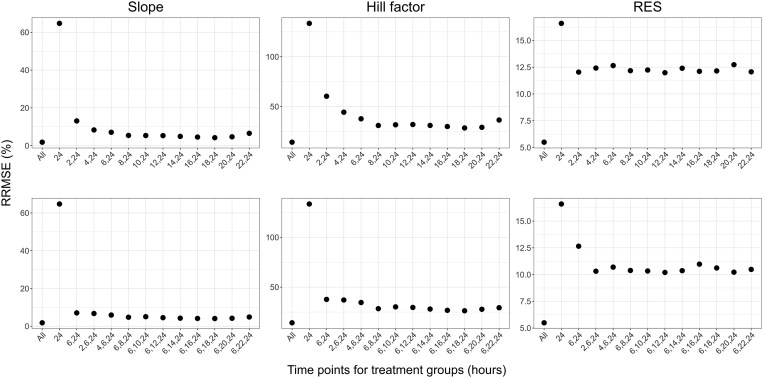
RRMSEs of parameter estimates for PMB for scenarios evaluating the inclusion of one (top row) or two (bottom row) extra time points (in hours after the start of treatment) for the evaluation of bacterial count in treatment groups. Each column presents RRMSEs for one parameter. Scenarios are indicated based on which time points were included in the design, with ‘All’ being the scenario with all evaluated time points and ‘24’ the simplest design with an effect evaluation at 24 h after the start of treatment only.

The distributions of REEs for the estimated parameters across the different designs are presented in Figure 4 for meropenem and Figure 5 for PMB. The RRMSEs of estimated parameters, under study designs with different treatment efficacy evaluation times, are presented in Figures 6 and 7, respectively. All RRMSE and relative bias values are reported in Tables S4 and S5.

As expected, the inclusion of all investigated time points led to the best parameter accuracies, with the overall lowest relative bias and RRMSE values and a reduced spread of REEs, while the lowest parameter accuracy was observed when only the typical 24-h time point was included in the analysis, resulting in the highest RRMSEs (e.g. RRMSE for PMB Slope of 1.84% and 64.7% for designs with all time-points and 24 h only, respectively). Slope and Shift estimates for meropenem had, in general, relatively low precision, including the design with all time-points.

All designs including one additional time point improved both bias and precision of meropenem Slope, Mut, and Shift parameter estimates compared to the 24-h-only design. In particular, additional time points in the early hours improved the accuracy of these parameters. With an additional measurement at 6 h, the Slope and Mut RRMSE reduced from 194% to 144%, and 397% to 56.6%, respectively. Slope and Shift parameters remained imprecise, although median parameter estimates were close to the true values. For PMB, the inclusion of any of the evaluated time points led to similar performances and improved the accuracy and precision for all parameters. The RRMSE for PMB Slope and γ were at least halved with the inclusion of one intermediate time point compared to a design with an evaluation of efficacy at 24 h only. Based on the RRMSEs, relative bias and distribution of REEs of parameter estimates, the first time point selected for designs evaluating two additional time points was 6 h after the start of treatment for both antibiotics.

The accuracy of parameter estimates for meropenem was further improved in all designs when adding a second intermediate time point after the start of treatment, where the design evaluating efficacy at 2, 6, and 24 h led to the best accuracy (e.g. RRMSE for Slope of 98.6% versus 91.6% with all time-points, and RRMSE for Shift of 1195% versus 1081% with all time-points). For PMB, adding a second time point did not significantly improve accuracy compared to the design with one additional time point.

## Discussion

This analysis explored the impact of dose fractionation study designs on antibiotic PKPD analyses using previously developed PKPD models for meropenem and PMB.^18–20^ By assessing the influence of various components of dose fractionation study designs, such as total daily doses and dosing intervals, on derived PK/PD indices, simulations provided further insights into the PK/PD index methodology and its limitations. Simulation and estimation analyses informed the potential use of dose fractionation studies for PKPD modelling approaches by identifying the additional bacterial count measurement time points that would most improve parameter estimation.

The models used in this analysis differ because they describe PKPD for antibiotics of different classes, typically associated with different PK/PD indices (*f*T > MIC for meropenem,^15^  *f*AUC/MIC for PMB).^16,17^ Moreover, the model structure applied for meropenem is more complex, describing resistance development over time. This allowed us to evaluate if specific design components could impact PKPD analyses for certain antibiotic classes more and if/how designs should be adapted to describe resistance processes better.

Simulations of various designs for a PK/PD index analysis indicated that the study design could affect both the selection of an index, with variations in *R*^2^ values, and the efficacy targets derived from PK/PD indices. With a rich design (Figure 1), *f*T > MIC was identified as the PK/PD index with the highest correlation for meropenem and *f*AUC/MIC for PMB, which aligns with earlier literature for mice.^15–17^ The derived efficacy targets were also of similar magnitudes as reported values.^17,30^ The PKPD models were thus able to replicate PK/PD indices accurately under a rich study design. Variations in *R*^2^ values were observed for the different designs, particularly with more frequent dosing intervals for meropenem and designs reducing the lower total dose for PMB, where *R*^2^ values for both *f*AUC/MIC and *f*T > MIC were similar. This suggests that PK/PD index analyses might be most affected by different elements of study design depending on the evaluated antibiotic. Improvements to *R*^2^ values of *f*C_max_/MIC were also observed despite a poor visual fit for *f*C_max_/MIC across all designs. Nonetheless, *f*T > MIC and *f*AUC/MIC were the indices with the highest correlations in most designs for meropenem and PMB, respectively. This confirms that selecting the ‘best’ PK/PD index can depend on the study design, as different indices could be chosen despite the simulations relying on the same underlying effect model. Furthermore, the typical selection of a PK/PD index through the *R*^2^ value has limitations, as this metric is not optimal for nonlinear data.^31^ It is also worth noting that MICs could further impact PK/PD indices. No discrepancy between *in vitro* MIC and *in vivo* susceptibility was included in simulations, even though MIC has known limitations, including biological and assay variability that influence the PK/PD index analysis.^5–7,14,32^ Although PK/PD indices are typically determined at 24 h after start of treatment, a determination at earlier time points may be valuable to save resources and reduce animal distress.

The magnitude of the PK/PD index required to reach efficacy targets was also sensitive to changes in the study design, especially the included dose range. For meropenem, *f*T > MIC targets increased with lower doses and decreased with higher doses: This is because *f*T > MIC depends on what point concentrations decrease below MIC while above MIC, it is not impacted by the concentration magnitude, i.e. to what extent the concentrations are higher than the MIC. For PMB, stasis and 1-log kill *f*AUC/MIC targets decreased with lower doses, and all PK/PD targets increased with higher doses, as a design where all doses are higher did not allow to characterize drug effect for lower AUCs. These discrepancies in PK/PD target determination may influence antibiotic dose selection, potentially exposing to a risk of toxicity or treatment failure. This underscores the importance of covering the dose-response curve in the study design to determine PK/PD targets accurately.

The SSEs performed for dose fractionation studies evaluated whether assessing efficacy at intermediate time points, in addition to an evaluation at 24 h of treatment, would be beneficial for PKPD modelling to describe the time course of antibiotic effect. As expected, including intermediate time points in the study design significantly improved the bias and/or precision of parameter estimates compared to model estimation based on only 24-h data, underscoring the importance of adapting dose fractionation designs for PKPD modelling. This was particularly visible with more complex underlying processes, with early time points required to describe resistance to meropenem. It should be noted that bacterial growth parameters for the strain to be used were assumed to be known. Growth control data should also be available at multiple time points to estimate those parameters adequately.^33^

For PMB, including one intermediate time point was sufficient to achieve acceptable accuracy of parameter estimates. Differences in parameter estimates between the evaluated additional times were limited, suggesting that the time point selection is less critical when the concentration-effect relationship is unlikely to vary significantly over the treatment period. However, lower accuracy was observed overall for meropenem, although median estimates were close to true values. This was primarily due to the concurrent estimation of both high slope and high shift in specific scenarios. This led to an exaggerated drug effect in susceptible bacteria and reduced potency in the resistant subpopulation. Estimation of model parameters may benefit from quantifying resistant bacteria in dose fractionation studies or from incorporating *in vitro* data.^33–35^ The inclusion of additional time points improved the accuracy of meropenem parameters. Early time points, notably 2 and 6 h after the start of treatment, appeared most beneficial as they allowed characterization of the initial decrease of bacterial counts before regrowth related to resistance occurred. These early times also offer the advantage of potentially avoiding overnight sampling. Additionally, later time points might be more likely to contain observations below the limit of quantification, limiting the information gained and potentially affecting the accuracy of parameter estimates. Given practical constraints associated with animal studies, time points were evaluated in 2-h steps. There may be unevaluated time points that provide a similar or slightly better degree of parameter accuracy. Optimal design methods may be employed on a case-by-case basis to refine study designs further. Although ethical concerns compel study designs to limit the number of animals, the information gained in experiments should also be maximized. Model-based approaches allow for an improved understanding of the antibiotic PKPD by describing the entire time course of the antibiotic effect. When adapting dose fractionation study designs for PKPD modelling, early evaluation of treatment efficacy, in addition to the usual 24-h endpoint, should be considered to enhance the reliability of the analysis while limiting the increase in study size. The suggested study designs allow for both the standard determination of PK/PD indices using the 24-h endpoint and the development of a PKPD model. Decision-making could thus be informed by derived PK/PD targets and predictions of bacterial killing performed using the PKPD model.

The analysis was based on a single PKPD model for each antibiotic. The sensitivity of the findings to the model structure was thus not tested. Other PKPD models, developed from different bacterial strains or antibiotics, may not necessarily provide the same optimal sampling points. The impact of the number of animals per group was not explored in the analysis, assuming that a sufficient number of animals per group would be included for results not to be affected by experimental variability. The uncertainty of the literature PKPD model parameters was also not considered in the simulations.

To conclude, simulations with several dose fractionation designs suggested that the choice of doses and dosing intervals may affect both the PK/PD index selection and the magnitude of derived efficacy targets. The choice of doses and the choice of dosing intervals appeared as the most impactful study design element for PMB and meropenem, respectively. The addition of measurements of antibiotic efficacy at 2 and 6 h after start of treatment was found to be valuable for PKPD model estimation, in particular to characterize resistance processes.

## Supplementary Material

dlaf057_Supplementary_Data

## Supplementary data

Tables S1–S5 is available as Supplementary data at *JAC-AMR* Online.
