# Supplementary material for: Simulation-based evaluation of the impact of dose fractionation study design on antibiotic PKPD analyses
Source: JAC Antimicrob Resist. 2025 Apr 11;7(2):dlaf057. doi: 10.1093/jacamr/dlaf057 (PMC11986329; doi:10.1093/jacamr/dlaf057)
Supplement: dlaf057_Supplementary_Data [file dlaf057_supplementary_data.docx]

**Table S1.** Parameters of the PK and PKPD models for meropenem and polymyxin B used for simulations.

| Antibiotic | Parameter (unit) | Description | Value |
| --- | --- | --- | --- |
| Meropenem | PK model | | |
|  | k_a_ (h^-1^) | Absorption rate constant | 10.2 |
|  | k_e_ (h^-1^) | Elimination rate constant | 2.36 |
|  | V/F (L/kg) | Apparent distribution volume | 0.566 |
|  | PKPD model | | |
|  | k_growth_ (h^-1^) | Bacterial growth rate constant | 1.08 |
|  | k_death_ (h^-1^) | Bacterial natural death rate constant | 0.179 |
|  | B_max_ (log_10_ cfu/mL) | Maximum bacterial count | 9.11 |
|  | Slope (L/mg•h) | Slope of bacterial killing due to meropenem | 2.16^a^ |
|  | γ (-) | Power parameter for meropenem effect | 0.376^a^ |
|  | Mut (%) | Fraction of pre-existing resistant bacteria | 0.15^a^ |
|  | Shift (-) | Shift in concentration required for effect on pre-existing resistant bacteria | 23.5^a^ |
|  | RES (log_10_ cfu/mL) | Additive residual error on the log_10_ scale | 0.422^a^ |
| Polymyxin B | PK model | | |
|  | k_a max_ (mg/h/kg) | Maximum absorption rate constant | 14.7 |
|  | k_a 50_ (mg/kg) | Amount in subcutaneous compartment that produces 50% of the maximum absorption rate | 2.24 |
|  | CL (L/h/kg) | Clearance | 0.437 |
|  | Vc (L/kg) | Distribution volume of the central compartment | 0.740 |
|  | Vp (L/kg) | Distribution volume of the peripheral compartment | 0.743 |
|  | CLd (L/h/kg) | Intercompartmental clearance | 0.315 |
|  | PKPD model | | |
|  | k_net_ (h^-1^) | Apparent growth rate constant | 0.594 |
|  | B_max_ (log_10_ cfu/thigh) | Maximum bacterial count in the tissue | 8.00 |
|  | Slope (L/mg•h) | Slope of bacterial killing due to polymyxin B | 1.00^a^ |
|  | γ (-) | Power parameter for polymyxin B effect | 0.162^a^ |
|  | RES (log_10_ cfu/thigh) | Additive residual error on the log_10_ scale | 1.63^a^ |

^a^Parameters re-estimated in the estimation step of SSEs.**Table S2.** Simulated dose fractionation study designs for the PK/PD index analysis of meropenem. The R² of the fits of the three PK/PD indices and the estimated PK/PD target values of *f*T>MIC required to reach stasis, 1-log kill and 2-log kill are reported for each simulated design.

|  |  |  | R² value | | | PK/PD target (*f*T>MIC %) | | |
| --- | --- | --- | --- | --- | --- | --- | --- | --- |
| Design | Doses (mg/kg/day) | Intervals (h) | *f*T>MIC | *f*Cmax/ MIC | *f*AUC/ MIC | Stasis | 1-log kill | 2-log kill |
| Rich | 25, 100, 200, 400, 600, 800, 1000, 1200 | 1, 2, 3, 4, 6, 8, 12, 24 | 0.877 | 0.132 | 0.465 | 25.1 | 29.2 | 35.0 |
| Literature | 200, 400, 800 | 3, 6, 12, 24 | 0.94 | 0.073 | 0.217 | 24.4 | 29.5 | 36.5 |
| Lower dose | 300, 400, 800 | 3, 6, 12, 24 | 0.966 | 0.077 | 0.186 | 23.3 | 28.0 | 34.5 |
|  | 250, 400, 800 |  | 0.956 | 0.074 | 0.195 | 23.8 | 28.7 | 35.4 |
|  | 150, 400, 800 |  | 0.918 | 0.075 | 0.265 | 25.0 | 30.3 | 37.7 |
|  | 100, 400, 800 |  | 0.877 | 0.083 | 0.345 | 25.8 | 31.7 | 40.1 |
|  | 80, 400, 800 |  | 0.872 | 0.089 | 0.386 | 26.4 | 32.4 | 40.5 |
|  | 50, 400, 800 |  | 0.86 | 0.265 | 0.455 | 27.1 | 32.7 | 40.0 |
|  | 25, 400, 800 |  | 0.942 | 0.489 | 0.51 | 24.3 | 27.6 | 32.0 |
| Higher dose | 200, 400, 2500 | 3, 6, 12, 24 | 0.798 | 0.145 | 0.414 | 21.2 | 26.5 | 34.4 |
|  | 200, 400, 2000 |  | 0.822 | 0.126 | 0.38 | 21.6 | 26.7 | 34.2 |
|  | 200, 400, 1600 |  | 0.853 | 0.116 | 0.342 | 22.1 | 27.2 | 34.6 |
|  | 200, 400, 1200 |  | 0.957 | 0.06 | 0.179 | 25.1 | 30.0 | 36.7 |
|  | 200, 400, 1000 |  | 0.919 | 0.085 | 0.253 | 23.7 | 28.9 | 36.2 |
|  | 200, 400, 600 |  | 0.957 | 0.06 | 0.179 | 25.1 | 30.0 | 36.7 |
|  | 200, 400, 500 |  | 0.967 | 0.053 | 0.158 | 25.6 | 30.5 | 37.3 |
|  | 100, 400, 500 |  | 0.909 | 0.058 | 0.271 | 26.9 | 32.5 | 40.4 |
|  | 80, 400, 500 |  | 0.904 | 0.061 | 0.31 | 27.5 | 33.1 | 40.8 |
| Middle dose | 200, 700, 800 | 3, 6, 12, 24 | 0.925 | 0.094 | 0.256 | 22.7 | 27.5 | 34.2 |
|  | 200, 600, 800 |  | 0.931 | 0.087 | 0.241 | 23.2 | 27.9 | 34.5 |
|  | 200, 450, 800 |  | 0.939 | 0.076 | 0.221 | 23.9 | 29.0 | 35.9 |
|  | 200, 350, 800 |  | 0.94 | 0.069 | 0.214 | 24.7 | 30.0 | 37.2 |
|  | 200, 250, 800 |  | 0.931 | 0.063 | 0.215 | 25.9 | 31.5 | 38.9 |
| Lower dose (double dose range) | 600, 800, 1600 | 3, 6, 12, 24 | 0.948 | 0.175 | 0.292 | 18.5 | 22.1 | 27.0 |
|  | 500, 800, 1600 |  | 0.94 | 0.166 | 0.294 | 18.8 | 22.5 | 27.5 |
|  | 400, 800, 1600 |  | 0.922 | 0.158 | 0.306 | 19.3 | 23.1 | 28.5 |
|  | 300, 800, 1600 |  | 0.896 | 0.15 | 0.328 | 19.6 | 23.7 | 29.6 |
|  | 200, 800, 1600 |  | 0.852 | 0.026 | 0.38 | 20.3 | 25.0 | 31.7 |
|  | 160, 800, 1600 |  | 0.834 | 0.151 | 0.431 | 20.7 | 25.5 | 32.7 |
|  | 100, 800, 1600 |  | 0.772 | 0.315 | 0.541 | 21.5 | 27.2 | 35.7 |
|  | 50, 800, 1600 |  | 0.751 | 0.629 | 0.648 | 22.7 | 28.6 | 36.9 |
| Lower dose (half dose range) | 100, 200, 400 | 3, 6, 12, 24 | 0.936 | 0.035 | 0.201 | 30.2 | 36.6 | 44.9 |
|  | 150, 200, 400 |  | 0.965 | 0.034 | 0.145 | 29.0 | 34.7 | 42.5 |
|  | 125, 200, 400 |  | 0.954 | 0.034 | 0.168 | 29.5 | 35.5 | 43.6 |
|  | 75, 200, 400 |  | 0.927 | 0.037 | 0.246 | 30.9 | 37.1 | 44.9 |
|  | 50, 200, 400 |  | 0.928 | 0.042 | 0.301 | 31.1 | 36.4 | 43.2 |
|  | 40, 200, 400 |  | 0.937 | 0.045 | 0.326 | 30.7 | 35.6 | 41.9 |
|  | 25, 200, 400 |  | 0.97 | 0.216 | 0.36 | 28.6 | 33.2 | 39.4 |
|  | 12.5, 200, 400 |  | 0.981 | 0.349 | 0.378 | 27.4 | 32.7 | 39.8 |
| Dosing interval | 200, 400, 800 | 6, 8, 12, 24 | 0.877 | 0.103 | 0.388 | 25.3 | 29.9 | 34.8 |
|  |  | 4, 8, 12, 24 | 0.915 | 0.082 | 0.275 | 24.3 | 28.5 | 34.4 |
|  |  | 4, 6, 12, 24 | 0.932 | 0.084 | 0.254 | 24.9 | 30.4 | 37.4 |
|  |  | 4, 6, 8, 24 | 0.927 | 0.119 | 0.315 | 27.3 | 30.7 | 35.1 |
|  |  | 4, 6, 8, 12 | 0.86 | 0.272 | 0.57 | 25.0 | 29.7 | 35.9 |
|  |  | 3, 8, 12, 24 | 0.933 | 0.069 | 0.23 | 24.2 | 27.9 | 33.0 |
|  |  | 3, 6, 8, 24 | 0.943 | 0.107 | 0.274 | 27.6 | 30.4 | 34.2 |
|  |  | 3, 6, 8, 12 | 0.94 | 0.073 | 0.217 | 24.4 | 29.5 | 36.5 |
|  |  | 2, 8, 12, 24 | 0.937 | 0.057 | 0.188 | 24.2 | 27.8 | 32.7 |
|  |  | 2, 6, 12, 24 | 0.942 | 0.061 | 0.203 | 24.3 | 29.3 | 36.3 |
|  |  | 2, 6, 8, 24 | 0.947 | 0.094 | 0.259 | 27.6 | 30.4 | 34.1 |
|  |  | 2, 6, 8, 12 | 0.882 | 0.227 | 0.469 | 24.9 | 29.2 | 35.0 |
|  |  | 2, 4, 12, 24 | 0.969 | 0.057 | 0.171 | 23.3 | 27.9 | 34.5 |
|  |  | 2, 4, 8, 24 | 0.966 | 0.092 | 0.225 | 27.0 | 29.0 | 31.6 |
|  |  | 2, 4, 8, 12 | 0.917 | 0.228 | 0.411 | 24.3 | 28.4 | 34.1 |
|  |  | 2, 4, 6, 12 | 0.919 | 0.271 | 0.441 | 24.6 | 29.7 | 36.8 |
|  |  | 1, 4, 8, 12 | 0.917 | 0.003 | 0.408 | 24.3 | 28.4 | 34.1 |
|  |  | 1, 3, 8, 12 | 0.934 | 0.198 | 0.372 | 24.2 | 27.8 | 32.8 |
|  |  | 1, 3, 6, 12 | 0.931 | 0.243 | 0.406 | 24.4 | 29.3 | 36.2 |
|  |  | 1, 4, 6, 8 | 0.903 | 0.45 | 0.668 | 27.4 | 30.6 | 35.0 |
|  |  | 1, 3, 6, 8 | 0.924 | 0.442 | 0.624 | 27.5 | 30.4 | 34.2 |
|  |  | 1, 2, 4, 6 | 0.966 | 0.344 | 0.795 | 31.2 | 33.7 | 36.9 |
|  |  | 1, 2, 3, 4 | 0.996 | 0.895 | 0.957 | 37.1 | 39.3 | 42.2 |
| Dose interval (double dose range) | 400, 800, 1600 | 6, 8, 12, 24 | 0.882 | 0.046 | 0.402 | 19.7 | 24.1 | 29.7 |
|  |  | 4, 8, 12, 24 | 0.893 | 0.171 | 0.346 | 19.6 | 23.6 | 29.0 |
|  |  | 4, 6, 12, 24 | 0.922 | 0.166 | 0.308 | 19.2 | 23.2 | 28.6 |
|  |  | 4, 6, 8, 24 | 0.944 | 0.199 | 0.323 | 19.4 | 24.0 | 30.3 |
|  |  | 4, 6, 8, 12 | 0.878 | 0.522 | 0.683 | 21.4 | 24.3 | 28.2 |
|  |  | 3, 8, 12, 24 | 0.893 | 0.162 | 0.344 | 19.6 | 23.6 | 29.0 |
|  |  | 3, 6, 8, 24 | 0.878 | 0.292 | 0.68 | 21.4 | 24.2 | 28.2 |
|  |  | 3, 6, 8, 12 | 0.943 | 0.192 | 0.322 | 19.1 | 23.6 | 29.9 |
|  |  | 2, 8, 12, 24 | 0.892 | 0.151 | 0.344 | 19.6 | 23.5 | 29.0 |
|  |  | 2, 6, 12, 24 | 0.922 | 0.148 | 0.306 | 19.3 | 23.1 | 28.4 |
|  |  | 2, 6, 8, 24 | 0.894 | 0.118 | 0.34 | 22.3 | 26.1 | 31.1 |
|  |  | 2, 6, 8, 12 | 0.878 | 0.126 | 0.68 | 21.4 | 24.2 | 28.2 |
|  |  | 2, 4, 12, 24 | 0.955 | 0.136 | 0.274 | 22.0 | 23.0 | 24.2 |
|  |  | 2, 4, 8, 24 | 0.95 | 0.171 | 0.297 | 18.8 | 23.2 | 29.5 |
|  |  | 2, 4, 8, 12 | 0.898 | 0.484 | 0.629 | 21.5 | 24.1 | 27.5 |
|  |  | 2, 4, 6, 12 | 0.97 | 0.523 | 0.633 | 21.9 | 22.9 | 24.3 |
|  |  | 1, 4, 8, 12 | 0.898 | 0.028 | 0.629 | 21.5 | 24.1 | 27.5 |
|  |  | 1, 3, 8, 12 | 0.916 | 0.434 | 0.597 | 21.4 | 24.3 | 28.3 |
|  |  | 1, 3, 6, 12 | 0.97 | 0.5 | 0.631 | 21.9 | 22.9 | 24.3 |
|  |  | 1, 4, 6, 8 | 0.969 | 0.746 | 0.869 | 27.9 | 29.4 | 31.3 |
|  |  | 1, 3, 6, 8 | 0.969 | 0.399 | 0.868 | 27.9 | 29.4 | 31.3 |
|  |  | 1, 2, 4, 6 | 0.999 | 0.949 | 0.968 | 33.3 | 34.4 | 35.9 |
|  |  | 1, 2, 3, 4 | 0.999 | 0.999 | 0.999 | 9.3 | 11.4 | 14.6 |
| Dose interval (half dose range) | 100, 200, 400 | 6, 8, 12, 24 | 0.914 | 0.056 | 0.373 | 29.0 | 32.8 | NR |
|  |  | 4, 8, 12, 24 | 0.938 | 0.038 | 0.244 | 30.3 | 36.1 | 42.4 |
|  |  | 4, 6, 12, 24 | 0.937 | 0.045 | 0.258 | 30.9 | 36.1 | 41.7 |
|  |  | 4, 6, 8, 24 | 0.928 | 0.07 | 0.338 | 30.3 | 35.2 | 40.9 |
|  |  | 4, 6, 8, 12 | 0.898 | 0.144 | 0.504 | 30.1 | 35.4 | 41.2 |
|  |  | 3, 8, 12, 24 | 0.947 | 0.028 | 0.18 | 30.1 | 37.0 | 46.0 |
|  |  | 3, 6, 8, 24 | 0.93 | 0.058 | 0.27 | 29.5 | 34.6 | 42.1 |
|  |  | 3, 6, 8, 12 | 0.904 | 0.117 | 0.401 | 29.3 | 35.1 | 43.2 |
|  |  | 2, 8, 12, 24 | 0.969 | 0.018 | 0.136 | 27.9 | 33.2 | 41.4 |
|  |  | 2, 6, 12, 24 | 0.957 | 0.024 | 0.158 | 29.3 | 35.0 | 43.4 |
|  |  | 2, 6, 8, 24 | 0.96 | 0.044 | 0.216 | 29.0 | 32.9 | 38.6 |
|  |  | 2, 6, 8, 12 | 0.936 | 0.094 | 0.323 | 28.7 | 33.6 | 40.8 |
|  |  | 2, 4, 12, 24 | 0.963 | 0.027 | 0.154 | 30.5 | 37.1 | 46.1 |
|  |  | 2, 4, 8, 24 | 0.956 | 0.049 | 0.211 | 30.0 | 36.0 | 44.5 |
|  |  | 2, 4, 8, 12 | 0.938 | 0.103 | 0.311 | 29.6 | 35.8 | 44.6 |
|  |  | 2, 4, 6, 12 | 0.925 | 0.137 | 0.371 | 30.5 | 36.0 | 43.5 |
|  |  | 1, 4, 8, 12 | 0.946 | 0.071 | 0.272 | 29.2 | 35.0 | 43.3 |
|  |  | 1, 3, 8, 12 | 0.955 | 0.079 | 0.245 | 29.2 | 35.7 | 45.3 |
|  |  | 1, 3, 6, 12 | 0.939 | 0.112 | 0.3 | 29.9 | 35.9 | 44.7 |
|  |  | 1, 4, 6, 8 | 0.924 | 0.197 | 0.474 | 30.0 | 34.6 | 41.1 |
|  |  | 1, 3, 6, 8 | 0.925 | 0.199 | 0.437 | 29.6 | 34.7 | 42.3 |
|  |  | 1, 2, 4, 6 | 0.925 | 0.189 | 0.616 | 31.6 | 35.8 | 41.8 |
|  |  | 1, 2, 3, 4 | 0.943 | 0.658 | 0.844 | 35.5 | 40.5 | 47.1 |

NR: Not reached.**Table S3.** Simulated dose fractionation study designs for the PK/PD index analysis of polymyxin B. The R² of the fits of the three PK/PD indices and the estimated PK/PD target values of *f*AUC/MIC required to reach stasis, 1-log kill and 2-log kill are reported for each simulated design.

|  |  |  | R² value | | | PK/PD target (*f*AUC/MIC) | | |
| --- | --- | --- | --- | --- | --- | --- | --- | --- |
| Design | Doses (mg/kg/day) | Intervals (h) | *f*T>MIC | *f*Cmax/ MIC | *f*AUC/ MIC | Stasis | 1-log kill | 2-log kill |
| Rich | 0.5, 10, 22.5, 30, 45, 60, 90, 120 | 1, 2, 3, 4, 6, 8, 12, 24 | 0.852 | 0.772 | 0.971 | 15.0 | 31.2 | 55.5 |
| Literature | 22.5, 45 ,90 | 4, 8, 12, 24 | 0.865 | 0.741 | 0.954 | 21.5 | 39.3 | 66.7 |
| Lower dose | 40, 45, 90 | 4, 8, 12, 24 | 0.891 | 0.769 | 0.954 | 25.7 | 45.5 | 74.7 |
|  | 35, 45, 90 |  | 0.876 | 0.761 | 0.954 | 24.2 | 43.5 | 72.6 |
|  | 30, 45, 90 |  | 0.862 | 0.752 | 0.954 | 23.5 | 42.4 | 71.0 |
|  | 20, 45, 90 |  | 0.869 | 0.740 | 0.955 | 20.7 | 38.0 | 65.0 |
|  | 15, 45, 90 |  | 0.912 | 0.746 | 0.956 | 18.8 | 35.0 | 60.8 |
|  | 10, 45, 90 |  | 0.943 | 0.771 | 0.958 | 16.4 | 31.3 | 55.5 |
|  | 8, 45, 90 |  | 0.948 | 0.790 | 0.959 | 15.3 | 29.5 | 53.0 |
|  | 4, 45, 90 |  | 0.915 | 0.850 | 0.964 | 13.2 | 26.6 | 49.1 |
|  | 2, 45, 90 |  | 0.941 | 0.895 | 0.972 | 16.7 | 35.6 | 64.4 |
|  | 1, 45, 90 |  | 0.961 | 0.919 | 0.981 | 19.5 | 39.4 | 69.3 |
|  | 0.75, 45, 90 |  | 0.965 | 0.925 | 0.983 | 20.1 | 40.0 | 69.9 |
|  | 0.5, 45, 90 |  | 0.969 | 0.931 | 0.986 | 20.9 | 40.7 | 70.5 |
| Middle dose | 22.5, 85, 90 | 4, 8, 12, 24 | 0.893 | 0.779 | 0.969 | 25.3 | 41.1 | 64.1 |
|  | 22.5, 80, 90 |  | 0.894 | 0.776 | 0.968 | 21.9 | 38.6 | 63.9 |
|  | 22.5, 75, 90 |  | 0.895 | 0.772 | 0.966 | 20.7 | 37.7 | 64.0 |
|  | 22.5, 70, 90 |  | 0.895 | 0.768 | 0.964 | 20.7 | 37.8 | 64.3 |
|  | 22.5, 65, 90 |  | 0.893 | 0.763 | 0.962 | 20.8 | 38.0 | 64.7 |
|  | 22.5, 85, 90 |  | 0.893 | 0.779 | 0.969 | 25.3 | 41.1 | 64.1 |
|  | 22.5, 55, 90 |  | 0.884 | 0.753 | 0.958 | 21.1 | 38.6 | 65.7 |
|  | 22.5, 50, 90 |  | 0.876 | 0.747 | 0.956 | 21.3 | 38.9 | 66.3 |
|  | 22.5, 40, 90 |  | 0.855 | 0.734 | 0.954 | 21.7 | 39.6 | 67.0 |
|  | 22.5, 35, 90 |  | 0.842 | 0.726 | 0.954 | 21.7 | 39.6 | 66.9 |
|  | 22.5, 30, 90 |  | 0.828 | 0.717 | 0.955 | 21.6 | 39.3 | 66.3 |
|  | 22.5, 25, 90 |  | 0.827 | 0.708 | 0.956 | 21.2 | 38.6 | 65.0 |
| Higher dose | 22.5, 45, 120 | 4, 8, 12, 24 | 0.811 | 0.738 | 0.961 | 22.9 | 41.0 | 67.6 |
|  | 22.5, 45, 115 |  | 0.819 | 0.738 | 0.960 | 22.4 | 40.4 | 67.3 |
|  | 22.5, 45, 110 |  | 0.827 | 0.738 | 0.959 | 22.3 | 40.4 | 67.5 |
|  | 22.5, 45,105 |  | 0.836 | 0.738 | 0.958 | 22.3 | 40.5 | 67.8 |
|  | 22.5, 45, 100 |  | 0.845 | 0.739 | 0.957 | 22.1 | 40.2 | 67.5 |
|  | 22.5, 45, 95 |  | 0.855 | 0.739 | 0.956 | 21.8 | 39.8 | 67.1 |
|  | 22.5, 45, 85 |  | 0.876 | 0.743 | 0.953 | 21.2 | 38.8 | 66.3 |
|  | 22.5, 45, 80 |  | 0.887 | 0.747 | 0.951 | 20.8 | 38.3 | 65.8 |
|  | 22.5, 45, 75 |  | 0.898 | 0.751 | 0.949 | 20.4 | 37.8 | 65.4 |
|  | 22.5, 45, 70 |  | 0.909 | 0.758 | 0.947 | 20.0 | 37.2 | 64.8 |
|  | 22.5, 45, 65 |  | 0.921 | 0.766 | 0.945 | 19.6 | 36.5 | 64.3 |
|  | 22.5, 45, 60 |  | 0.932 | 0.776 | 0.943 | 19.1 | 35.9 | 63.7 |
|  | 22.5, 45, 55 |  | 0.943 | 0.789 | 0.941 | 19.3 | 35.7 | 63.3 |
|  | 22.5, 45, 50 |  | 0.952 | 0.784 | 0.938 | 26.6 | 40.8 | 63.4 |
| Lower dose (double dose range) | 45, 90, 120 | 4, 8, 12, 24 | 0.883 | 0.809 | 0.970 | 33.1 | 53.8 | 82.2 |
|  | 80, 90, 120 |  | 0.975 | 0.867 | 0.973 | 43.8 | 67.1 | 97.2 |
|  | 70, 90, 120 |  | 0.960 | 0.925 | 0.972 | 43.4 | 66.6 | 96.5 |
|  | 60, 90, 120 |  | 0.936 | 0.897 | 0.971 | 40.0 | 62.5 | 92.0 |
|  | 40, 90, 120 |  | 0.868 | 0.801 | 0.971 | 30.6 | 50.7 | 78.6 |
|  | 30, 90, 120 |  | 0.842 | 0.793 | 0.972 | 25.6 | 44.1 | 70.8 |
|  | 20, 90, 120 |  | 0.877 | 0.799 | 0.975 | 20.7 | 37.4 | 62.4 |
|  | 16, 90, 120 |  | 0.923 | 0.810 | 0.976 | 19.1 | 34.7 | 58.4 |
|  | 8, 90, 120 |  | 0.986 | 0.890 | 0.981 | 14.9 | 27.4 | 47.0 |
|  | 4, 40, 120 |  | 0.958 | 0.943 | 0.985 | 11.7 | 21.8 | 38.1 |
|  | 2, 90, 120 |  | 0.975 | 0.969 | 0.988 | 9.5 | 18.0 | 31.8 |
|  | 1.5, 90, 120 |  | 0.981 | 0.976 | 0.989 | 8.9 | 16.8 | 29.9 |
|  | 1, 90, 120 |  | 0.987 | 0.982 | 0.990 | 8.2 | 15.7 | 28.0 |
| Lower dose (half dose range) | 20, 22.5, 45 | 4, 8, 12, 24 | 0.943 | 0.784 | 0.923 | 17.8 | 34.1 | 62.9 |
|  | 17.5, 22.5, 45 |  | 0.943 | 0.758 | 0.924 | 17.4 | 33.5 | 62.2 |
|  | 15, 22.5, 45 |  | 0.953 | 0.732 | 0.926 | 16.9 | 32.7 | 61.3 |
|  | 11.25, 22.5, 45 |  | 0.963 | 0.699 | 0.930 | 15.8 | 31.0 | 59.3 |
|  | 10, 22.5, 45 |  | 0.964 | 0.693 | 0.932 | 15.4 | 30.3 | 58.6 |
|  | 7.5, 22.5, 45 |  | 0.966 | 0.695 | 0.937 | 14.3 | 28.7 | 56.7 |
|  | 5, 22.5, 45 |  | 0.958 | 0.723 | 0.943 | 13.0 | 26.7 | 54.6 |
|  | 4, 22.5, 45 |  | 0.906 | 0.743 | 0.947 | 12.5 | 25.9 | 53.7 |
|  | 2, 22.5, 45 |  | 0.935 | 0.807 | 0.957 | 12.2 | 26.2 | 54.6 |
|  | 1, 22.5, 45 |  | 0.964 | 0.853 | 0.968 | 14.5 | 30.7 | 60.1 |
|  | 0.5, 22.5, 45 |  | 0.977 | 0.881 | 0.975 | 15.9 | 32.6 | 62.0 |
|  | 0.375, 22.5, 45 |  | 0.980 | 0.889 | 0.976 | 16.2 | 33.0 | 62.3 |
| Dose interval | 22.5, 45, 90 | 6, 8, 12, 24 | 0.874 | 0.782 | 0.959 | 21.9 | 40.0 | 67.9 |
|  |  | 4, 6, 12, 24 | 0.848 | 0.730 | 0.951 | 21.2 | 38.7 | 65.8 |
|  |  | 4, 6, 8, 24 | 0.837 | 0.734 | 0.950 | 20.6 | 37.7 | 64.1 |
|  |  | 4, 6, 8, 12 | 0.849 | 0.878 | 0.991 | 19.4 | 35.3 | 59.5 |
|  |  | 3, 8, 12, 24 | 0.865 | 0.719 | 0.951 | 21.3 | 38.9 | 66.1 |
|  |  | 3, 6, 12, 24 | 0.848 | 0.712 | 0.949 | 20.9 | 38.3 | 65.2 |
|  |  | 3, 6, 8, 24 | 0.836 | 0.718 | 0.948 | 20.4 | 37.4 | 63.5 |
|  |  | 3, 6, 8, 12 | 0.847 | 0.846 | 0.989 | 19.2 | 34.9 | 59.0 |
|  |  | 2, 8, 12, 24 | 0.865 | 0.700 | 0.949 | 21.0 | 38.5 | 65.5 |
|  |  | 2, 6, 12, 24 | 0.848 | 0.698 | 0.946 | 20.7 | 38.0 | 64.6 |
|  |  | 2, 6, 8, 24 | 0.836 | 0.705 | 0.946 | 20.2 | 37.0 | 63.0 |
|  |  | 2, 6, 8, 12 | 0.846 | 0.815 | 0.988 | 19.0 | 34.6 | 58.5 |
|  |  | 2, 4, 12, 24 | 0.869 | 0.712 | 0.941 | 19.0 | 35.4 | 61.5 |
|  |  | 2, 4, 8, 24 | 0.829 | 0.701 | 0.942 | 19.9 | 36.4 | 61.9 |
|  |  | 2, 4, 8, 12 | 0.850 | 0.821 | 0.987 | 18.4 | 33.7 | 57.3 |
|  |  | 2, 4, 6, 12 | 0.815 | 0.815 | 0.988 | 18.5 | 33.7 | 56.9 |
|  |  | 1, 4, 8, 12 | 0.833 | 0.790 | 0.986 | 18.6 | 33.9 | 57.3 |
|  |  | 1, 3, 8, 12 | 0.831 | 0.787 | 0.985 | 18.4 | 33.6 | 56.8 |
|  |  | 1, 3, 6, 12 | 0.814 | 0.797 | 0.986 | 18.2 | 33.2 | 56.1 |
|  |  | 1, 4, 6, 8 | 0.834 | 0.832 | 0.993 | 18.0 | 32.8 | 55.3 |
|  |  | 1, 3, 6, 8 | 0.830 | 0.830 | 0.992 | 17.9 | 32.5 | 54.9 |
|  |  | 1, 2, 4, 6 | 0.845 | 0.862 | 0.994 | 17.3 | 31.5 | 53.1 |
|  |  | 1, 2, 3, 4 | 0.896 | 0.898 | 0.996 | 16.9 | 30.8 | 52.0 |
| Dose interval (double dose range) |  | 6,8,12,24 | 0.881 | 0.856 | 0.974 | 34.0 | 55.1 | 83.8 |
|  |  | 4,6,12,24 | 0.885 | 0.795 | 0.968 | 32.5 | 53.0 | 81.0 |
|  |  | 4,6,8,24 | 0.887 | 0.788 | 0.968 | 31.8 | 51.8 | 79.3 |
|  |  | 4,6,8,12 | 0.869 | 0.918 | 0.995 | 31.7 | 50.7 | 76.2 |
|  |  | 3,8,12,24 | 0.884 | 0.774 | 0.968 | 32.5 | 53.1 | 81.3 |
|  |  | 3,6,12,24 | 0.886 | 0.763 | 0.967 | 32.0 | 52.3 | 80.1 |
|  |  | 3,6,8,24 | 0.888 | 0.767 | 0.966 | 31.3 | 51.2 | 78.4 |
|  |  | 3,6,8,12 | 0.870 | 0.878 | 0.994 | 31.3 | 50.2 | 75.5 |
|  |  | 2,8,12,24 | 0.885 | 0.738 | 0.967 | 31.9 | 52.3 | 80.3 |
|  |  | 2,6,12,24 | 0.887 | 0.730 | 0.965 | 31.4 | 51.5 | 79.2 |
|  |  | 2,6,8,24 | 0.889 | 0.733 | 0.965 | 30.7 | 50.4 | 77.5 |
|  |  | 2,6,8,12 | 0.871 | 0.830 | 0.993 | 30.9 | 49.6 | 74.8 |
|  |  | 2,4,12,24 | 0.888 | 0.712 | 0.962 | 30.6 | 50.4 | 77.8 |
|  |  | 2,4,8,24 | 0.891 | 0.715 | 0.962 | 30.1 | 49.5 | 76.2 |
|  |  | 2,4,8,12 | 0.872 | 0.814 | 0.993 | 30.4 | 48.8 | 73.7 |
|  |  | 2,4,6,12 | 0.874 | 0.818 | 0.993 | 30.1 | 48.3 | 73.0 |
|  |  | 1,4,8,12 | 0.876 | 0.777 | 0.992 | 29.9 | 48.2 | 73.1 |
|  |  | 1,3,8,12 | 0.877 | 0.851 | 0.992 | 29.6 | 47.8 | 72.4 |
|  |  | 1,3,6,12 | 0.879 | 0.900 | 0.992 | 29.4 | 47.4 | 71.8 |
|  |  | 1,4,6,8 | 0.860 | 0.876 | 0.996 | 29.5 | 47.4 | 71.4 |
|  |  | 1,3,6,8 | 0.861 | 0.896 | 0.996 | 29.2 | 47.0 | 70.9 |
|  |  | 1,2,4,6 | 0.863 | 0.904 | 0.997 | 28.5 | 45.7 | 69.0 |
|  |  | 1,2,3,4 | 0.866 | 0.806 | 0.998 | 28.1 | 45.0 | 67.9 |
| Dose interval (half dose range) | 11.25, 22.5, 45 | 6,8,12,24 | 0.970 | 0.733 | 0.938 | 16.1 | 31.6 | 60.4 |
|  |  | 4,6,12,24 | 0.955 | 0.690 | 0.926 | 15.6 | 30.5 | 58.4 |
|  |  | 4,6,8,24 | 0.952 | 0.695 | 0.924 | 15.1 | 29.6 | 56.5 |
|  |  | 4,6,8,12 | 0.956 | 0.887 | 0.988 | 14.1 | 27.4 | 51.1 |
|  |  | 3,8,12,24 | 0.956 | 0.682 | 0.925 | 15.6 | 30.7 | 58.8 |
|  |  | 3,6,12,24 | 0.949 | 0.676 | 0.921 | 15.4 | 30.2 | 57.8 |
|  |  | 3,6,8,24 | 0.945 | 0.683 | 0.920 | 15.0 | 29.3 | 56.0 |
|  |  | 3,6,8,12 | 0.950 | 0.859 | 0.986 | 14.0 | 27.1 | 50.6 |
|  |  | 2,8,12,24 | 0.940 | 0.667 | 0.921 | 15.5 | 30.4 | 58.3 |
|  |  | 2,6,12,24 | 0.934 | 0.664 | 0.917 | 15.2 | 29.9 | 57.3 |
|  |  | 2,6,8,24 | 0.931 | 0.671 | 0.917 | 14.8 | 29.0 | 55.5 |
|  |  | 2,6,8,12 | 0.935 | 0.829 | 0.983 | 13.9 | 26.9 | 50.2 |
|  |  | 2,4,12,24 | 0.928 | 0.656 | 0.912 | 14.9 | 29.3 | 56.3 |
|  |  | 2,4,8,24 | 0.926 | 0.665 | 0.912 | 14.5 | 28.5 | 54.5 |
|  |  | 2,4,8,12 | 0.930 | 0.822 | 0.982 | 13.6 | 26.4 | 49.3 |
|  |  | 2,4,6,12 | 0.925 | 0.834 | 0.983 | 13.5 | 26.0 | 48.6 |
|  |  | 1,4,8,12 | 0.905 | 0.802 | 0.981 | 13.5 | 26.2 | 49.1 |
|  |  | 1,3,8,12 | 0.900 | 0.795 | 0.979 | 13.4 | 26.0 | 48.7 |
|  |  | 1,3,6,12 | 0.896 | 0.808 | 0.980 | 13.3 | 25.7 | 47.9 |
|  |  | 1,4,6,8 | 0.898 | 0.872 | 0.991 | 13.1 | 25.3 | 47.0 |
|  |  | 1,3,6,8 | 0.894 | 0.866 | 0.990 | 13.0 | 25.1 | 46.6 |
|  |  | 1,2,4,6 | 0.880 | 0.907 | 0.993 | 12.6 | 24.2 | 44.8 |
|  |  | 1,2,3,4 | 0.888 | 0.948 | 0.996 | 12.3 | 23.7 | 43.7 |

**Table S4**. RRMSE (in %) of the estimated model parameters depending on the CFU measurement time points included for treatment groups in the dose fractionation study design.

|  | Meropenem parameters | | | | | Polymyxin B parameters | | |
| --- | --- | --- | --- | --- | --- | --- | --- | --- |
| Time points (h) | Slope | γ | Mut | Shift | RES | Slope | γ | RES |
| All | 91.6 | 2.18 | 24.1 | 1081 | 5.16 | 1.84 | 14.2 | 5.50 |
| 24 | 194 | 5.16 | 397 | 2346 | 16.8 | 64.7 | 133 | 16.6 |
| 2, 24 | 113 | 5.09 | 174 | 1274 | 13.1 | 13.1 | 60.3 | 12.0 |
| 4, 24 | 167 | 5.10 | 115 | 2072 | 12.4 | 8.32 | 44.3 | 12.4 |
| 6, 24 | 144 | 5.02 | 56.6 | 1793 | 12.2 | 7.08 | 37.7 | 12.6 |
| 8 ,24 | 155 | 4.84 | 70.0 | 1838 | 12.5 | 5.41 | 31.0 | 12.2 |
| 10, 24 | 158 | 4.33 | 56.7 | 1919 | 12.5 | 5.36 | 31.7 | 12.2 |
| 12, 24 | 153 | 4.06 | 49.0 | 1849 | 12.6 | 5.30 | 32.0 | 12.0 |
| 14, 24 | 180 | 4.29 | 75.3 | 2215 | 12.5 | 4.90 | 31.0 | 12.4 |
| 16, 24 | 179 | 3.90 | 91.7 | 2163 | 12.7 | 4.54 | 29.9 | 12.1 |
| 18, 24 | 171 | 3.66 | 85.0 | 2074 | 12.5 | 4.22 | 28.5 | 12.1 |
| 20, 24 | 182 | 3.88 | 103 | 2200 | 12.7 | 4.70 | 29.1 | 12.7 |
| 22, 24 | 180 | 3.89 | 134 | 2136 | 12.2 | 6.53 | 36.5 | 12.1 |
| 2, 6, 24 | 98.6 | 4.94 | 57.8 | 1195 | 10.4 | 6.76 | 37.1 | 10.3 |
| 4, 6, 24 | 138 | 4.80 | 53.9 | 1682 | 10.1 | 5.93 | 34.6 | 10.7 |
| 6, 8, 24 | 143 | 4.64 | 47.4 | 1746 | 9.82 | 4.78 | 28.4 | 10.4 |
| 6, 10, 24 | 148 | 4.46 | 41.3 | 1779 | 10.3 | 5.05 | 30.2 | 10.3 |
| 6, 12, 24 | 134 | 4.05 | 42.0 | 1614 | 10.6 | 4.54 | 29.6 | 10.2 |
| 6, 14, 24 | 141 | 4.02 | 49.0 | 1706 | 10.1 | 4.27 | 28.0 | 10.4 |
| 6, 16, 24 | 141 | 3.73 | 51.8 | 1718 | 10.4 | 4.13 | 26.7 | 11.0 |
| 6, 18, 24 | 137 | 3.49 | 47.7 | 1669 | 9.93 | 4.08 | 26.3 | 10.6 |
| 6, 20, 24 | 134 | 3.46 | 45.8 | 1666 | 10.3 | 4.22 | 27.8 | 10.2 |
| 6, 22, 24 | 126 | 3.87 | 50.3 | 1463 | 10.5 | 4.89 | 29.3 | 10.5 |

**Table S5.** Relative bias (in %) of the estimated model parameters depending on the CFU measurement time points included for treatment groups in the dose fractionation study design.

|  | Meropenem parameters | | | | | Polymyxin B parameters | | |
| --- | --- | --- | --- | --- | --- | --- | --- | --- |
| Time points (h) | Slope | γ | Mut | Shift | RES | Slope | γ | RES |
| All | 33.7 | -0.17 | 0.93 | 368 | -0.64 | 0.09 | 1.14 | -0.14 |
| 24 | 115 | -0.09 | 93.6 | 1542 | -6.94 | 7.39 | 23.0 | -1.71 |
| 2, 24 | 44.2 | 0.11 | 55.6 | 478 | -3.04 | 3.16 | 12.8 | -0.63 |
| 4, 24 | 92.0 | -0.65 | 15.7 | 1166 | -4.08 | 1.40 | 5.38 | -1.90 |
| 6, 24 | 67.7 | -0.75 | -3.84 | 877 | -1.83 | 1.22 | 5.86 | -1.57 |
| 8 ,24 | 74.0 | -0.27 | 3.66 | 952 | -3.21 | -0.19 | -0.80 | -2.40 |
| 10, 24 | 78.5 | -0.43 | 0.14 | 1029 | -2.49 | 0.66 | 3.23 | -0.80 |
| 12, 24 | 71.3 | -0.40 | -1.99 | 951 | -3.35 | 0.07 | 1.25 | -1.24 |
| 14, 24 | 103 | -0.40 | 8.66 | 1365 | -2.95 | 0.48 | 1.34 | -1.78 |
| 16, 24 | 100 | -0.22 | 15.9 | 1315 | -2.75 | 0.06 | 0.03 | -1.72 |
| 18, 24 | 94.8 | -0.23 | 9.17 | 1245 | -2.35 | -0.48 | -0.32 | -1.54 |
| 20, 24 | 105 | -0.05 | 22.5 | 1369 | -2.65 | 0.46 | 2.12 | -1.35 |
| 22, 24 | 98.9 | -0.03 | 27.2 | 1300 | -2.98 | 0.61 | 3.21 | -1.76 |
| 2, 6, 24 | 37.0 | -0.91 | 5.92 | 420 | -2.51 | 0.20 | -1.47 | -1.09 |
| 4, 6, 24 | 65.6 | -0.44 | -3.04 | 803 | -2.28 | 0.50 | 0.72 | -1.38 |
| 6, 8, 24 | 68.9 | -0.73 | -1.35 | 851 | -2.25 | -0.02 | 0.48 | -1.21 |
| 6, 10, 24 | 73.3 | -0.34 | -1.55 | 900 | -1.97 | 0.49 | 1.22 | -0.30 |
| 6, 12, 24 | 60.0 | -0.17 | 0.52 | 744 | -1.45 | 0.09 | 0.48 | -1.76 |
| 6, 14, 24 | 69.3 | -0.75 | -1.73 | 847 | -2.20 | 0.38 | 2.66 | -1.65 |
| 6, 16, 24 | 67.8 | -0.56 | 2.26 | 842 | -1.25 | -0.01 | 0.48 | -1.56 |
| 6, 18, 24 | 64.9 | -0.37 | 1.62 | 802 | -2.60 | -0.04 | -0.44 | -1.13 |
| 6, 20, 24 | 59.7 | -0.60 | -5.87 | 773 | -0.95 | 0.28 | 0.74 | -0.04 |
| 6, 22, 24 | 52.7 | -0.61 | -2.58 | 648 | -1.82 | 0.16 | 0.61 | -1.55 |
